# HIV infection and arterial inflammation assessed by ^18^F-fluorodeoxyglucose (FDG) positron emission tomography (PET): A prospective cross-sectional study

**DOI:** 10.1007/s12350-014-0032-0

**Published:** 2014-12-03

**Authors:** Andreas Knudsen, Anne Mette Fisker Hag, Annika Loft, Eric von Benzon, Sune H. Keller, Holger Jon Møller, Anne-Mette Lebech, Rasmus Sejersten Ripa, Andreas Kjær

**Affiliations:** 1 Department of Infectious Diseases, Copenhagen University Hospital, Hvidovre, Kettegaard Allé 30, 2650 Hvidovre, Denmark; 2 Department of Clinical Physiology, Nuclear Medicine & PET, Copenhagen University Hospital, Rigshospitalet and Cluster for Molecular Imaging, Faculty of Health and Medical Sciences, University of Copenhagen, Copenhagen, Denmark; 3 Department of Clinical Biochemistry, Aarhus University Hospital, Aarhus, Denmark

**Keywords:** Fluorodeoxyglucose (FDG), PET/CT imaging, inflammation

## Abstract

**Background:**

HIV-infected patients are at increased risk of myocardial infarction and arterial inflammation has been suggested as a pathophysiological explanation. We compared the uptake of ^18^F-fluorodeoxyglucose (FDG) by PET in four arterial regions, and factors associated with FDG uptake in well-treated HIV-infected patients without cardiovascular disease (CVD) and healthy controls.

**Methods and Results:**

We prospectively scanned 26 HIV-infected patients on stable antiretroviral therapy and 25 healthy volunteers with FDG PET/CT, measuring standardized uptake values (SUV) in the carotid arteries, the ascending, descending, and abdominal aorta. We performed correlation analyses between FDG uptake and intima-media thickness (IMT), and soluble biomarkers of inflammation. We found no difference in arterial FDG uptake between the HIV-infected patients and healthy controls quantified either as mean SUV_max_ or target-to background ratio in the carotid region, the ascending aorta, the descending aorta, or the abdominal aorta. Correlations between SUV, IMT, and soluble biomarkers were scarce in both groups.

**Conclusion:**

In a group of optimally treated HIV-infected patients with full viral suppression, low Framingham risk score and no known CVD, we found no evidence of increased arterial inflammation as assessed by FDG PET/CT compared to healthy volunteers.

## Introduction

HIV-infected patients are at increased risk of myocardial infarction (MI)^1^ and subclinical coronary atherosclerosis even when low HIV RNA levels and high CD 4 cell counts are obtained with antiretroviral therapy (ART),^2^-^4^ however, the mechanism behind remains to be fully elucidated. Both the HIV infection itself and the ART have been shown to cause chronic inflammation and endothelial dysfunction,^5^-^7^ associated with upregulation of adhesion molecules marking the beginning of an atherosclerotic lesion.^8^ In HIV-infected patients these lesions seem to be less calcified^3^,^9^ making them more vulnerable and prone to rupture.^10^ Macrophages are known to play a key role in the development of atherosclerosis^11^ and recently a possible mechanistic role has been ascribed to the macrophage in causing the increased risk^12^-^14^ among HIV-infected patients.

The activated macrophage has a high metabolic rate and is therefore readily visualized by positron emission tomography (PET) using the glucose analog ^18^F-fluorodeoxyglucose (FDG).^15^ If increased inflammatory activity is present in arteries of HIV-infected we would expect an increased FDG uptake compared to the arteries in comparable healthy controls. Studies of this very early atherosclerosis would provide knowledge of the pathogenesis behind the increased risk of cardiovascular disease (CVD) among HIV-infected patients with full viral suppression and may also offer ways to monitor early atherosclerosis and guide future interventions against CVD in this population.

We conducted a cross-sectional, prospective study of 26 HIV-infected patients and 25 healthy controls using FDG PET/CT with the concomitant measurement of intima-media thickness (IMT), soluble biomarkers of inflammation, macrophage activation, and endothelial dysfunction.

## Methods

### Participants

A total of 53 persons were scanned between March 2011 and June 2013.

Twenty-six HIV-infected patients were prospectively recruited at routine visits at the out-patient clinic at the department of infectious diseases, Copenhagen University Hospital, Hvidovre, Denmark.

Inclusion criteria were HIV-infection, male sex, age > 18 years, and ART > 12 months and exclusion criteria were known CVD (myocardial infarction, stable/unstable angina, arrhythmia, peripheral arterial disease, or stroke), diabetes mellitus, renal insufficiency, or use of lipid lowering drugs.

A total of 27 healthy volunteers were recruited prospectively through online advertising using the same exclusion criteria.

Of the 27 healthy volunteers 2 were excluded due to the incidental finding of a minor thymoma and pulmonary cancer on the PET/CT-scans, respectively.

### Ethics

All patients received oral and written information and gave written consent before inclusion. The study was approved by the Scientific Ethics Committee of The Capital Region of Denmark [protocol number H-4-2010-044] and complied with the declaration of Helsinki.

### PET/CT-Scans

After an overnight fast, 400 MBq (10.8 mCi) FDG was injected by auto-injector, and to reduce tracer uptake in neck and mouth muscles and brown fat, the patients were not allowed to talk and rested in calm and warm surroundings from 15 minutes before injection to 30 minutes after injection.

Three hours after injection, the scan was performed on a combined PET/CT-scanner (Siemens Biograph mCT64, Siemens, Erlangen, Germany). Each participant underwent a CT-scan from the *arcus zygomaticus* to the *crista iliaca* for attenuation correction and after the PET-scan a CT-scan with intravenous contrast (Optiray^®^) in arterial phase for better visualization of the carotid arteries. The CT-scan used 120 kV, 225 mAs (care dose). Slice thickness was 2 mm.

The PET-scan was performed with 3-4 bed positions depending of the height of the patient covering the area from the *arcus zygomaticus* to the *crista iliaca*. PET acquisition time was 3 minutes per bed position in 3D list mode. The system used both resolution-recovery (point spread function, TrueX), time-of-flight, and OSEM (2 iterations, 21 subsets, zoom 1.0) giving 400 × 400 image slices (voxel size 2.04 × 2.04 × 2.00 mm).

### PET/CT Image Reconstruction

**Figure 1:**
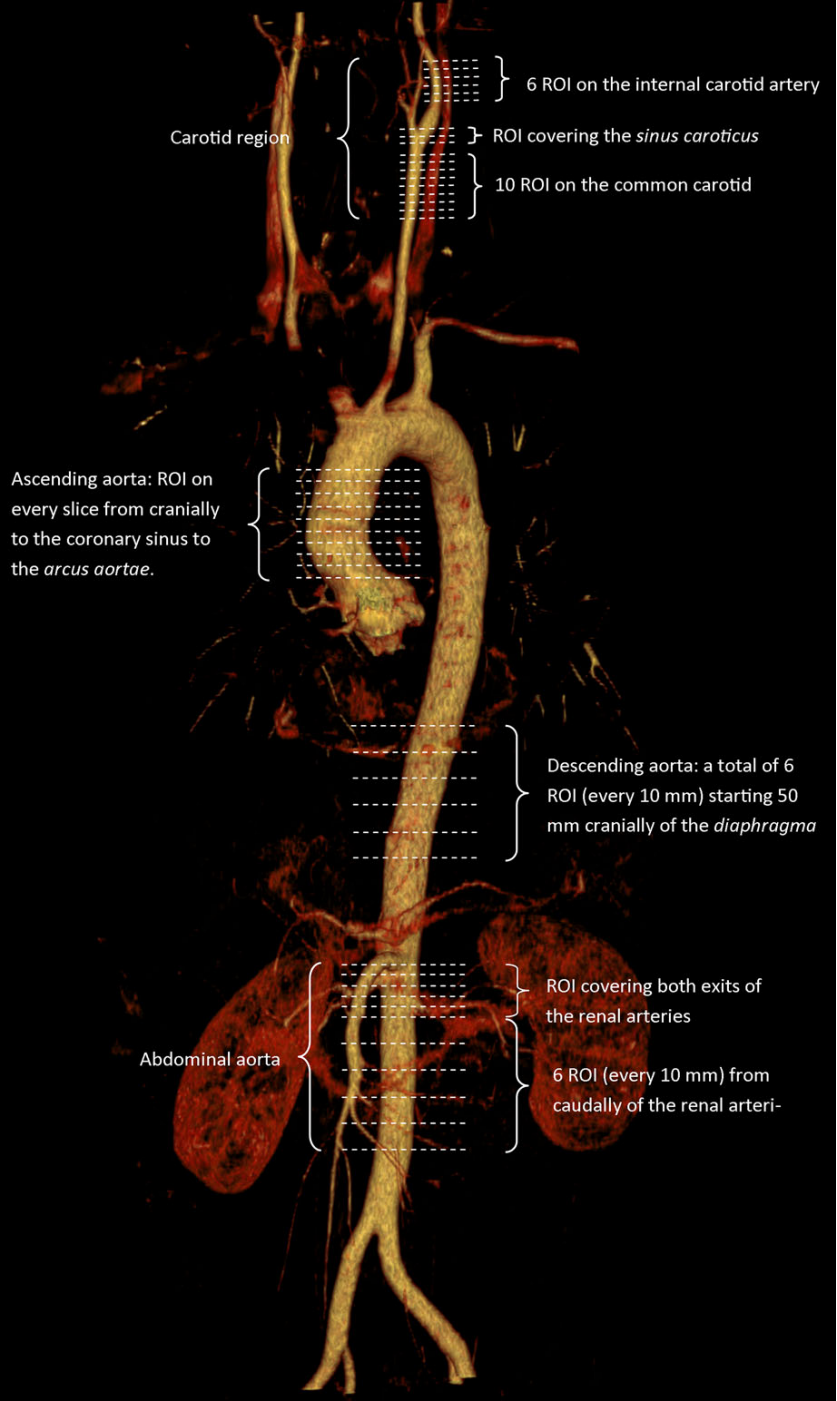
CT-angiography showing the areas of the arterial tree where regions of interest (ROI) were drawn for the analysis of FDG uptake. Slice thickness on CT was 2 mm, but distances on this image are not to actual scale

Four anatomical areas were chosen per protocol for analysis: the carotid arteries (*a. carotis interna*, *sinus caroticus*, and *a. carotis communis*), the ascending aorta (*pars ascendens aortae*—cranially to the coronary sinus), the descending aorta (*pars thoracica aortae*), and the abdominal aorta (*pars abdominalis aortae*). For the correlation analysis of the IMT and FDG uptake a mean SUV_max_ for the common carotid artery on both sides was calculated (Figure 1).

Image analysis was performed on Inveon™ Research Workplace (Siemens, Erlangen, Germany) by the same analyst using a standard protocol. Regions of interest (ROI) were manually drawn slice by slice including both vessel wall and lumen. Standardized uptake values (SUV) were calculated by dividing the decay-corrected radioactivity concentration (MBq·L^−1^) by the injected radioactive dose per body weight (MBq·kg^−1^) assuming a mass density of body tissue of 1 kg·L^−1^.

For each region a mean arterial SUV_max_ was calculated. The target-to-background ratio (TBR) was calculated by dividing the mean SUV_max_ by the mean SUV from the *vena cava superior*.

### Plasma Markers

Plasma samples were analyzed using commercially available kits according to the manufacturer’s instructions. High sensitivity C-reactive protein (hsCRP) was measured on a Siemens STRATUS CS in duplicate with a coefficient of variance (CV) of 8.6%.

Soluble CD163 (sCD163) was measured by an in-house sandwich enzyme-linked immunosorbent assay using a BEP-2000 ELISA-analyser (Dade Behring, Deerfield, IL, USA).^16^

Soluble endothelial selectin (sE-selectin), soluble vascular cell adhesion molecule1 (sVCAM-1), soluble intercellular adhesion molecule 1 (sICAM-1), matrix metalloprotease 9 (MMP-9), and total plasminogen activator inhibitor 1 (PAI-1) were analyzed on a multiplex assay (Milipore Corporation, MA, USA) using Luminex^®^ technology.

All samples were run in duplicates with CV < 12%. CD4 cell counts and HIV RNA levels were done routinely on blood and plasma when collected. Serum lipids were analyzed on a MODULAR ISE 1800 (Roche, Basel, Switzerland).

Metabolic syndrome was defined as having at least two of the following: fasting blood glucose ≥ 5.6 mmol·L^−1^ or (systolic blood pressure ≥ 135 mm Hg or diastolic blood pressure ≥ 85 mm Hg) or (triglycerides > 1.7 mmol·L^−1^ or high density lipoprotein <1.03 mmol·L^−1^).^17^

Framingham risk score (FRS) was calculated as the 10-year risk of coronary heart disease (CHD) according to published definitions.^18^

### Intima-Media Thickness

Before tracer injection, real-time IMT was measured in the common carotid artery 1 cm caudally of the *sinus caroticus* by ultrasound (Mylab25Gold, Esaote, Italy) using a 6.5 MHz transducer and automatic software (RF-QIMT, Esaote, Italy).

### Statistics

Data are reported as mean values ± standard error of the mean.

The HIV-infected patients and the healthy controls were compared using unpaired *t* test. Variables were tested for normal distribution using the Kolmogorov-Smirnov-test and log-transformed to obtain a normal distribution and analyzed by parametric test. Correlations were analyzed using Spearman’s *ρ* on untransformed data.

The *adjusted* unpaired *t*-test was performed by multiple linear regression, and test for interaction was performed with a general linear model.

We performed a Bonferroni correction for multiple comparisons in the correlation analysis, and we therefore considered a *p*-value of .0125 significant.

With a total of 51 patients in the two groups we obtained a power of 0.8 to detect a difference of ~18% in FDG uptake (alpha 0.05) using previously published standard deviation on SUV_max_ from arteries.^19^

One healthy control did not have blood drawn and another healthy control was not scanned over the neck region. Two healthy controls had missing data on blood pressure.

All statistics were performed using SPSS 20 (IBM SPSS statistics for windows, version 20.0 Armonk, NY, IBM Corp).

## Results

The characteristics of the HIV-infected group and healthy controls are shown in Table 1.

**Table 1:** Baseline characteristics

	HIV-infected		Healthy controls		*p* value*
	Mean	95% CI	Mean	95% CI	
Number	26		25		
Male gender	26	(100)	25	(100)	
Age (years)	50.5	45.5-55.5	41.2	35.8-46.5	.01
Tobacco use					
Current, *n* (%)	5	(19)	7	(28)	.23^†^
Former, *n* (%)	11	(42)	5	(20)	
Never, *n* (%)	10	(39)	13	(52)	
Statin use	0		0		
Hemodynamics					
Systolic blood pressure (mm Hg)	134	128-141	127	122-133	.09
Diastolic blood pressure (mm Hg)	82	78-86	74	70-78	.004
Risk calculations					
Framingham 10 yr CHD risk (%)	7.8	5.4-10.2	4.1	2.1-6-1	.03
Lipid profile					
Total cholesterol (mmol·L^−1^)	5.3	4.8-5.9	4.6	4.0-5.1	.03
HDL (mmol·L^−1^)	1.6	1.3-1.8	1.5	1.4-1.7	.63
LDL (mmol·L^−1^)	3.2	2.8-3.6	2.8	2.2-3.3	.18
TG (mmol·L^−1^)	1.3	1.0-1.6	1.1	0.9-1.2	.14
Blood glucose (mmol·L^−1^)	5.4	5.1-5.7	4.9	4.7-5.1	.01
BMI	24.0	22.9-25.1	24.8	23.7-26.0	.28
HIV parameters					
CD4 cell count (10^6^)·L^−1^	636	549-717			
HIV duration (years)	13.9	10.8-16.8			
ART duration (years)	9.9	8-11.7			
HIV RNA copies/mL (median, range)	19	19-31			
≥2 NRTIs + 1 NNRTI (%)	21	(81)			
≥2 NRTIs + ≥1 PI (%)	4	(15)			
Other (%)	1	(4)			

*Unpaired *t*-test. ^†^ *χ* ^2^-test

*ART*, antiretroviral therapy; *BMI*, body mass index; *CHD*, coronary heart disease; *HDL*, high density lipoprotein; *LDL*, low density lipoprotein; *NNRTI*, non-nucleoside reverse transcriptase inhibitor; *NRTI*, nucleoside reverse transcriptase inhibitor; *PI*, protease inhibitor; *TG*, triglycerides

The HIV-infected group was older (50.5 ± 2.4 vs 41.2 ± 2.6 yrs; *p* = .01) and had higher blood pressure and total cholesterol, and accordingly a higher FRS. Three HIV-infected patients and 1 healthy control had metabolic syndrome.

### HIV-Parameters

The HIV-infected group was stable on ART with a mean CD 4 cell count of 636 (10^6^/L), and all had viral loads <40 copies/mL. A total of 21/25 (81%) of the HIV-infected individuals received a regimen of 2 nucleoside reverse transcriptase inhibitors (NRTIs) and 1 non-nucleoside reverse transcriptase inhibitor (NNRTI), whereas 15% received a regimen including a protease inhibitor (PI) (Table 1).

### Markers of Endothelial Dysfunction and Inflammation

**Figure 2:**
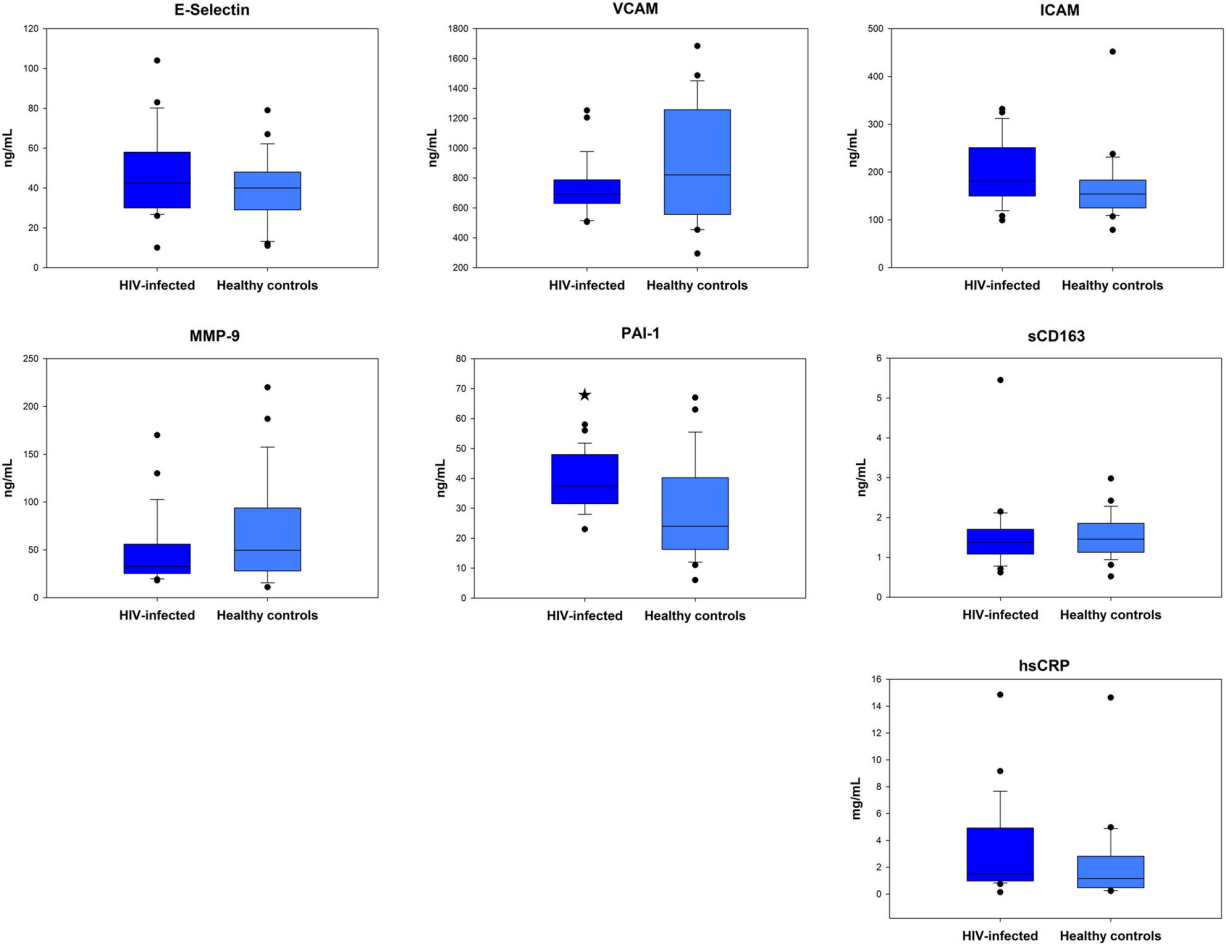
Plasma levels of soluble biomarkers. Abbreviations: *sCD163*, soluble cluster of differentiation 163. *sE-selectin*, soluble endothelial selectin. *hsCRP*, high sensitivity C-reactive protein. *sICAM-1*, soluble intercellular adhesion molecule 1. *MMP-9*, matrix metalloprotease 9. *PAI-1*, plasminogen activator inhibitor 1. *sVCAM-1* soluble vascular cell adhesion molecule 1. *Boxes* represent 25%-75% intervals and whiskers represent 90% confidence intervals. *Asterisk* indicates *p* < 0.05

The plasma level of the coagulation marker, PAI-1, was significantly higher in the HIV-infected group than among healthy controls (38.6 ± 1.8 vs 28.7 ± 3.3 ng·mL^−1^; *p* = .009). However, the levels of soluble biomarkers of inflammation (hsCRP), activated macrophages (CD163), and endothelial dysfunction (E-Selectin, VCAM-1, ICAM-1, MMP-9) did not differ between the two groups (Figure 2).

### Inter Group Differences in Arterial Wall FDG Uptake

**Figure 3:**
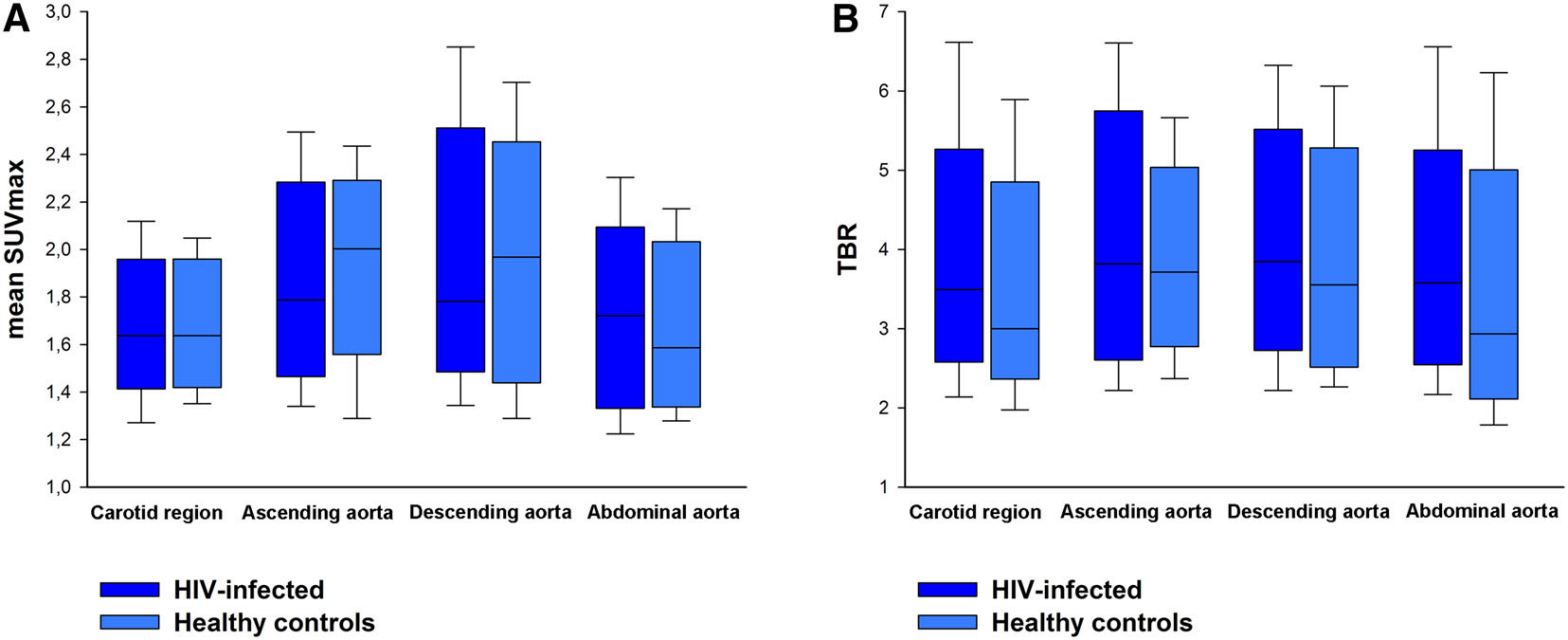
**A** Mean SUVmax in the four regions for HIV-infected and healthy controls. **B** Target-to-background ratio (TBR) in the four regions for HIV-infected and healthy controls. *Boxes* represent 25%-75% intervals and *whiskers* represent 90% confidence intervals

**Figure 4:**
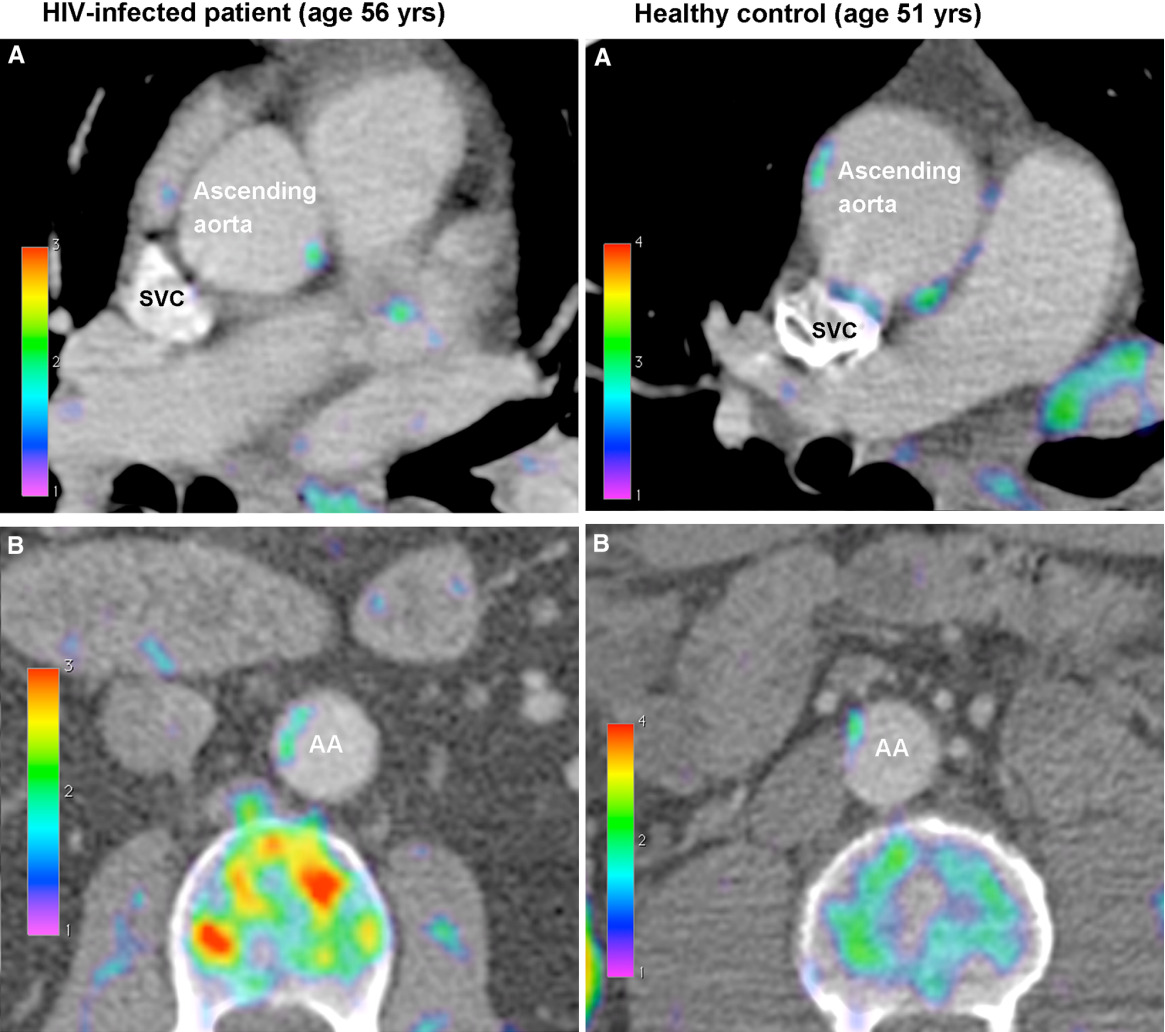
Fusion PET/CT axial images from the ascending (*panel A*) and abdominal aorta (*panel B*) of an HIV-infected patient and a healthy control showing modest FDG uptake in the arterial wall. *AA*, abdominal aorta; *FDG*, fluorodeoxyglucose; *SVC*, superior vena cava

The two groups displayed no significant differences in mean SUV_max_ in the carotid region (1.67 ± 0.04 vs 1.67 ± 0.04, *p* = .98), the ascending aorta (1.84 ± 0.06 vs 1.97 ± 0.06, *p* = .15), the descending aorta (1.89 ± 0.08 vs 1.93 ± 0.08, *p* = .70), or the abdominal aorta (1.70 ± 0.06 vs 1.65 ± 0.06, *p* = .56), HIV-infected vs healthy controls, respectively. The two groups also displayed similar values of TBR in the four regions: 3.59 ± 0.20 vs 3.25 ± 0.19, *p* = .16 in the carotid region; 4.00 ± 0,25 vs 3.77 ± 0.17, *p* = .64 in the ascending aorta; 4.04 ± 0,23 vs 3.71 ± 0.21, *p* = .31 in the descending aorta; and 3.64 ± 0,20 vs 3.24 ± 0.23, *p* = .11 in the abdominal aorta (Figures 3A, B, 4), and these findings remained unchanged when adjusting the analysis for the variables by which the groups were significantly different, *i.e*., age, systolic blood pressure, diastolic blood pressure, total cholesterol, and blood glucose (Data not shown).

### Intima-Media Thickness

No difference was found between the two groups in mean IMT on the right (630 ± 26 vs 634 ± 21 µm; *p* = .78), and the left (674 ± 26 vs 636 ± 25 µm; *p* = .29), or in a mean value of the two sides (652 ± 24 vs 635 ± 18 µm; *p* = .67), HIV-infected vs healthy controls, respectively. The values remained without statistical difference when adjusting for the variables by which the groups differed, *i.e*., age, systolic blood pressure, diastolic blood pressure, total cholesterol, and blood glucose (Data not shown).

There was no correlation between IMT and FDG uptake in the right (*ρ* = −0.115; *p* = .58) or left (*ρ* = 0.398; *p* = .05) common carotid artery among HIV-infected or healthy controls (*ρ* = −0.141; *p* = .51), and (*ρ* = 0.261; *p* = .22), right and left common carotid artery, respectively.

### HIV Parameters and FDG Uptake

CD4 cell count was inversely correlated to FDG uptake in the descending aorta (*ρ* = −0.707; *p* < .0001), whereas no correlations were found between duration of HIV-infection, duration of ART or HIV RNA viral load, and FDG uptake.

### Biomarkers and FDG Uptake

In the HIV-infected group there were no correlations between the soluble biomarkers of inflammation and FDG uptake beside an inverse correlation between sCD163 and FDG uptake in the descending aorta (*ρ* = −0.517; *p* = .007). None were found in the healthy control group.

As the only biomarker to be significantly different between the two groups, PAI-1 was analyzed by a multiple regression model and found independently associated with FDG uptake in the carotid region and the descending aorta (*β* = 0.224; *p* = .048) and (*β* = 0.506; *p* = .006), respectively.

There was no interaction between HIV-status and the soluble biomarkers.

## Discussion

There is a well-established link between macrophages and CVD, and with the correlation between ^18^F-FDG PET and macrophages,^20^ PET/CT-scan could possibly be of use in the assessment of arterial inflammation as sign of early CVD, which is of concern among HIV-infected individuals.^14^ However, in this cross-sectional study of 51 individuals, we did not find a difference in FDG uptake in the arterial wall of four different regions of the arterial tree between HIV-infected and healthy controls. This result was surprising in the light of previous studies of FDG uptake in HIV-infected patients. First, Yarasheski et al performed a proof-of-concept study among nine HIV-infected patients with known cardiovascular risk factors and five healthy volunteers and found proof of vascular inflammation in the HIV-infected group, where eight out of nine patients had evidence of carotid plaque.^21^ Another and larger study by Subramanian et al found that HIV-infected patients without CVD had higher FDG uptake in the ascending aorta than a matched healthy control group with the conclusion that HIV-infection was associated with arterial inflammation. Further, this inflammation was found to be associated with sCD163 among the HIV-infected patients making macrophages the most likely suspects behind the increased FDG uptake.^14^

Our study design differed in many ways from the study by Subramanian et al first and foremost, we prospectively included all participants to the exact same imaging methodology on a single PET/CT-system, and our PET examinations were therefore all performed 3 hours after FDG injection. The importance of similar FDG circulation time was recently emphazised in several studies.^19^,^22^ Especially the TBR has been shown to increase over time due to the washout of FDG in the lumen of the vessel (background), which happens faster than the decay in the arterial wall.^19^,^22^,^23^ Therefore, comparison of TBR values between HIV-infected patients scanned after 3 hours with individuals scanned after 60 minutes as per-clinical (oncological) protocol 24 as done in the trial by Subramanian et al could potentially produce false high values among the HIV-infected individuals.^14^

Our study population was very similar to the participants included in the study by Subramanian et al, but a few potentially important differences exist. Their HIV-infected group had >40% of patients currently treated with PI vs 15% in our study, and since PI have been shown to affect endothelial function this may have an impact on the uptake of FDG.^25^

Moreover, we included no individuals treated with statins, which are proven to have anti-inflammatory effects and may have attenuated the FDG tracer uptake 26 among the healthy controls.

Our comparison of soluble biomarkers of inflammation, macrophages activation and endothelial dysfunction as well as IMT supports our FDG results in showing very few differences between optimally treated HIV-infected patients with high CD 4 counts and full viral suppression and healthy controls. The only soluble biomarker that differed between the groups was the marker of anti-fibrinolytic activity, PAI-1, which was significantly higher in the HIV-infected group. Interestingly, PAI-1 was also independently associated with FDG uptake in two vascular regions in the HIV-infected group. We have recently found an association between increased levels of PAI-1 and the risk of MI among HIV-infected patients,^27^ but whether the association between PAI-1 levels and FDG uptake found in this study constitutes a causal link between PAI-1 and CVD remains to be elucidated.

The groups did not differ with regard to IMT, and the correlation analysis of FDG uptake and IMT did not show any significance. Indeed, both groups had IMT levels within normal range.^28^

Our group of HIV-infected patients was carefully selected not to have any known CVD or diabetes, which was reflected in low FRS and normal IMT. This may impair the ability to detect correlations with FDG uptake since the association of FDG and macrophages is most strongly shown in lesions from more advanced atherosclerosis.^29^,^30^ Further a recent ex vivo study suggests that hypoxia rather than the presence of macrophages is correlated with FDG uptake.^31^ Taken together, our data do imply that optimally treated HIV-infection in patients with no known CVD and low FRS does not cause arterial inflammation as assessed by biomarkers, IMT, or FDG uptake.

However, it should be emphasized that conclusions from this well-treated HIV-infected population do not nescessarily extrapolate to non-selected populations, where risk factors may be more prevalent.

## Limitations

The sample size of 51 subjects is relatively small, but had ample power to detect differences in FDG tracer uptake in the aforementioned studies with FDG. The small sample size limits the study’s ability to detect differences between other parameters and as such more subtle differences may have gone undetected while others could have arisen by chance alone. Our two groups were not perfectly matched and differences in risk profile had to be adjusted for in the statistical analyses. However, this difference would, if anything, make the HIV-infected group more likely to have arterial inflammation. To make full use of our scans we included data from the descending and abdominal aorta although a study showed higher interscan variability in these areas compared to the carotid arteries and the ascending aorta.^32^

## New Knowledge Gained

In this study we found no evidence of arterial inflammation among *optimally* treated HIV-infected patients with low cardiovascular risk and no known CVD.

## Conclusion

In a group of HIV-infected patients on stable ART with full viral suppression, no known CVD and low FRS we did not find evidence of arterial inflammation as assessed by FDG uptake in four different regions of the arterial wall compared to a group of healthy volunteers. This may indicate that well-treated HIV-infection *per se* is not associated with arterial inflammation.
